# A model for professional nurses to facilitate culturally sensitive nursing care for children

**DOI:** 10.4102/curationis.v49i1.2867

**Published:** 2026-08-04

**Authors:** Catherine Mngomezulu, Charlene Downing

**Affiliations:** 1 Department of Nursing, Faculty of Health Sciences, University of Johannesburg, Johannesburg, South Africa

**Keywords:** children, cultural care model, culturally sensitive nursing care, facilitation, model development, professional nurse

## Abstract

**Background:**

Culturally sensitive nursing care is vital in paediatric settings, where children’s health experiences and responses to care are influenced by their cultural beliefs, family practices and developmental needs.

**Objectives:**

This study aimed to develop, describe and evaluate a model to guide professional nurses in providing culturally sensitive care for hospitalised children.

**Method:**

A theory-generative, qualitative, exploratory, descriptive and contextual design was employed. The model was created through four systematic steps: concept analysis, formulation of relationship statements, model construction and expert evaluation.

**Results:**

The resulting model helps professional nurses to recognise and clarify their own values, incorporate institutional cultural guidelines and utilise relevant resources to facilitate culturally sensitive care.

**Conclusion:**

At the core of the model is the active involvement of mothers or primary caregivers throughout the child’s hospitalisation, ensuring holistic, culturally responsive and developmentally appropriate care.

**Contribution:**

The model provides a practical framework to improve cultural competence and enhance paediatric nursing care across diverse clinical settings.

## Introduction

### Background

Cultural care in nursing was introduced by Madeleine Leininger in the mid-1950s to address patients’ cultural needs (Leininger 1988). Culture continues to shape people’s experiences of healthcare (Öner et al. 2023) as people often interpret healthcare according to their specific cultural beliefs (Shopo et al. 2023). Culture forms part of the children’s upbringing, and children are especially influenced by culture, as it shapes their worldview. It is important for the professional nurse to know the cultural beliefs and practices of the children they nurse (Dertli & Gunay 2022). Culture remains intrinsic to who people are and how they make healthcare decisions (Collins et al. 2025). It is therefore important for professional nurses to be sensitive to cultural practices to improve the quality of care and life (Öner et al. 2023).

Over the years, there has been an increase in cultural diversity owing to global migration. Professional nurses are exposed to patients from diverse cultural backgrounds. Professional nurses are expected to provide culturally sensitive nursing care (SANC 2022); however, they often lack the frame of reference to guide them in providing such care. The model, therefore, aims at assisting professional nurses to facilitate culturally sensitive nursing care throughout the children’s hospitalisation journey. Because of the increasing complexity of healthcare, including evolving cultural diversity, there is a need to develop new models to guide nursing practice (Andrews & Boyles 2019).

Cultural care models and frameworks have been developed over the years to address patients’ cultural care needs (Collins et al. 2025); however, they do not focus on the unique needs of the children. While these adult-focused models made significant contributions to the nursing practice regarding cultural care, they do not respond to the children’s unique developmental needs and the importance of the family’s involvement in the care of the child. The lack of a dedicated child-centred cultural care model limits professional nurses’ ability to facilitate holistic and inclusive care to children. Children need the presence of their mothers or primary caregivers during hospitalisation to provide familiarity similar to the home environment (Mabetha, De Wet-Billings & Odimegwu 2021). Literature suggests that cultural beliefs and practices influence the perceptions and choices of healthcare services (Mabetha et al. 2021). Mabetha et al. (2021) believes that the involvement of primary caregivers, including mothers, in the care of children has a positive influence on the children’s outcomes because their presence makes the children feel more secure and provides a sense of normalcy akin to the home environment. Despite this evidence, current models do not integrate primary caregivers’ involvement as a central component of paediatric nursing care. The model provides a guiding framework for professional nurses to deliver holistic care that is responsive to family dynamics and needs, children’s developmental stages and various cultural practices, which is its unique contribution to South African paediatric nursing. The model aims to incorporate the active and direct involvement of the mother or the primary caregiver in the child’s hospitalisation journey, recognising their indispensable role in the child’s well-being. In doing so, it not only facilitates holistic, culturally sensitive, child-centred nursing care, but it also promotes family- and community-centred care essential for a healthy ecosystem. The model also enforces collaboration between the healthcare professional, the family and the community.

Provision of culturally sensitive nursing care requires professional nurses to master the skills of cultural competence (Dertly & Gunay 2022), which is the ability to facilitate care within the patient’s context (Leininger 1988; Öner et al. 2023). The study answers the following research question: ‘*What can be done to facilitate culturally sensitive nursing care for children?*’ The model further aims to empower professional nurses with cultural competence skills. Campinha-Bacote (2002) identified five skills of cultural competence: cultural awareness, cultural knowledge, cultural skill, cultural encounter and cultural desire. The model provides professional nurses with guidelines to apply these skills to provide culturally appropriate and sensitive care.

### Theoretical framework

Several theories guide the model. It is theoretically grounded in Leininger’s Culture Care Theory and Sunrise Model, as well as Campinha-Bacote’s Model of Cultural Competence. Dickoff, James and Wiedenbach (1968) and Chinn, Kramer and Sitzman (2022) provide the model with its structure and rigour. The model is tied together by the Ubuntu philosophy, highlighting mutual respect (Muhammad-Lawal et al. 2023) and the connectedness between the professional nurse and the recipients of care. Peplau’s Theory of Interpersonal Relationships (1997) provides the structural pathway for the children’s hospitalisation journey in three phases: relationship phase, working phase and termination phase. The three phases coincide with the patients’ admission, hospitalisation and discharge, respectively. During admission, which coincides with the relationship phase, the professional nurse leads the engagement and invites the mother to share cultural care expectations. This builds trust and brings them together to collaborate in care.

During the hospital stay, which coincides with the working phase, there is a deeper connection between the professional nurse, the child and the mother. The professional nurse shows deep compassion through this collaboration. Throughout hospitalisation, the professional nurse engages with the recipients of care with trust and mutual understanding, and this brings them closer together for effective facilitation of culturally sensitive nursing care. In the final phase of the model – the termination phase, which coincides with discharge the professional nurse, the child and the mother together engage in a reflection of care. Peplau’s three phases of interpersonal relationships enhance cultural care by promoting meaningful engagement.

### Study purpose

The research purpose was to develop, describe and evaluate a model to enable professional nurses to facilitate culturally sensitive practices for the nursing care of children at a children’s hospital. The study was conducted in two phases: descriptive phenomenology and model development. The model was developed in four steps: Step 1: Concept analysis; Step 2: Relationship statements; Step 3: Model development and description and Step 4: Model evaluation (Chinn et al. 2022).

## Research methods and design

### Study design

A theory-generative, qualitative, exploratory, descriptive and contextual design was used to develop a model for professional nurses to facilitate culturally sensitive practices in nursing care for children at a children’s hospital. The study was conducted in two phases. In the first phase, a qualitative, exploratory, descriptive and contextual design was used to explore the experiences of professional nurses and mothers of sick children regarding culturally sensitive nursing care. These phenomenological experiences were used as a foundation to develop the model in the second phase. The second phase employed a theory-generative design.

### Study methods

#### Trustworthiness

To ensure trustworthiness, the study employed credibility, transferability, dependability and confirmability as outlined by Amin et al. (2020). Credibility was enhanced by exploring the lived experiences of the professional nurses and mothers, allowing for an in-depth understanding of cultural sensitivity. Transferability was enhanced through a detailed description of the study context, participants, data collection and analysis procedures. Furthermore, an audit trail of the research process was carried out to ensure dependability by documenting all the stages of the research process. The data were analysed independently by the researcher, supervisor and an independent coder to ensure confirmability, thereby minimising researcher bias and ensuring that the findings were grounded in the collected data.

The study was conducted in two phases: a descriptive phenomenological approach and model development. Data for the descriptive phenomenological phase were collected from July 2023 to May 2024. Data analysis was followed from May 2025 to August 2024. The findings were then synthesised in preparation for the model development. The model was developed in four steps: Step 1: Concept analysis from September 2024 to December 2024, Step 2: Relationship statements from January 2025 to February 2025, Step 3: Model development from March 2025 to August 2025 and Step 4: Model evaluation in August 2025.

#### Phase 1: Descriptive phenomenological approach

The first phase of the study used a qualitative, exploratory, descriptive and contextual design to describe the experiences of culturally sensitive nursing care from the perspectives of the carers, professional nurses and the recipients of care, mothers of sick children in line with Husserlian descriptive phenomenology. The researcher prevented their own bias by engaging in bracketing to suspend their preconceived ideas. Furthermore, the researcher had prolonged engagement with the study participants and constant debriefing with the supervisor (Amin et al. 2020). The findings from this descriptive phenomenological study yielded a central concept: ‘facilitation of culturally sensitive practices for nursing care’. A children’s hospital in Gauteng, South Africa, was utilised as the study context. The study comprised two participant populations: (1) professional nurses and (2) mothers of children admitted to the children’s hospital because of illness. The demographic details of the participants are outlined in Table 1 and Table 2, respectively. Both participant groups had diverse cultural backgrounds. Semi-structured, in-depth, individual interviews were conducted and audio-recorded with participants’ consent. The participants were asked two central questions: (1) ‘*What are your experiences of culturally sensitive nursing care?*’ and (2) ‘*What is your understanding of culture?*’. The second question was introduced following the pilot study. Reflective and observational field notes were taken before, during and immediately after each interview to record additional information to substantiate the findings. Data saturation was reached after interviewing 12 professional nurses and seven mothers. At these points, the data were redundant, and the participants were no longer providing new information.

**TABLE 1:** Demographic characteristics of Group 1 participants, professional nurses.

Participants	Age (years)	Gender	Ethnicity	Year of joining the hospital
Participant 1 (PNP 1)	36	Female	Swati person	2021
Participant 2 (PNP 2)	34	Female	Xhosa person	2019
Participant 3 (PNP 3)	35	Female	Tsonga person	2017
Participant 4 (PNP 4)	38	Female	Pedi person	2019
Participant 5 (PNP 5)	31	Female	Pedi person	2018
Participant 6 (PNP 6)	44	Male	Pedi person	2018
Participant 7 (PNP 7)	33	Female	Tswana person	2019
Participant 8 (PNP 8)	27	Female	Tswana person	2021
Participant 9 (PNP 9)	29	Female	Pedi person	2021
Participant 10 (PNP 10)	27	Female	Coloured person	2018
Participant 11 (PNP 11)	29	Female	Sotho person	2019
Participant 12 (PNP 12)	41	Female	Zulu person	2017

**TABLE 2:** Demographic characteristics of Group 2 participants, primary caregivers of the admitted children.

Participants	Age (years)	Gender	Ethnicity	Relationship to the admitted child
Participant 1 (MP 1)	36	Female	Venda person	Mother
Participant 2 (MP 2)	43	Female	Tswana person	Mother
Participant 3 (MP 3)	33	Female	Zulu person	Mother
Participant 4 (MP 4)	23	Female	Venda person	Mother
Participant 5 (MP 5)	46	Female	Pedi person	Mother
Participant 6 (MP 6)	40	Female	Tswana person	Mother
Participant 7 (MP 7)	51	Female	Pedi person	Mother

Data were analysed using Braun and Clarke’s thematic analysis approach (Braun & Clarke 2006) (Table 3). The interviews were transcribed verbatim. Three themes emerged from the professional nurse participants: (1) tensions between professional responsibility and cultural sensitivity, (2) dilemma of misunderstood expectations and (3) need for awareness of and formal guidelines for acceptable cultural practices.

**TABLE 3:** Thematic analysis using Braun and Clarke.

Steps of thematic analysis	Activities
Familiarisation with the data	The audio-recorded data were transcribed verbatim and read repeatedly to get familiar with the findings
Generating initial codes	Initial codes expressed by the participants were listed
Searching for themes	Similar and related codes were grouped together
Reviewing themes	The themes were reviewed by three people
Defining and naming themes	The three people defined and agreed about the final themes
Producing the report	The report was produced by providing the themes and supporting quotes from the participants

Balancing advocacy role and cultural sensitivity is often frustrating as the professional nurses feel overpowered. One participant reported:

‘Others will tell you: I believe this is a case of witchcraft. I still want to go home and appease the ancestors. And it becomes an issue because the traditional medicines are not scientifically tested. What I can only say to the parents is these are the findings from the doctor. They say the child needs cardiac surgery which is an emergency. The more you delay, the child’s condition might worsen. As health professionals you advise but at the end of the day, it remains with the parents to make a decision.’ (PNP 6, 44 year old, male)

The professional nurses grappled with balancing cultural sensitivity and their professional duty to protect the children. Professional nurses also believe that the cultural misunderstandings could be resolved by understanding that the cultural beliefs are not the same:

‘I think as nurses, we need to understand that our cultural beliefs are not the same and we need to accommodate them.’ (PNP 12, 41 year old, female)

and

‘So, you have to understand the [the] kind of parents that you are nursing and where they are coming from.’ (PNP 1, 36 year old, female)

Professional nurses acknowledged having gaps in cultural knowledge, underscoring the need for institutional policies to support culturally sensitive nursing care. One professional nurse conceded:

‘I think we need education. I think we don’t know about certain things. So the reason why nurses … the reason why managers, doctors will not allow certain things is because they don’t understand.’ (PNP 11, 29 year old, female)

Two themes emerged from the mothers: (1) disregard for certain cultural practices and (2) cultural misunderstandings. One participant expressed frustration over the disregard as follows:

‘I feel like it takes away my right to take care of my child in any kind of way which I feel like it as a parent.’ (MP 1, 36 year old, female, Mother)

One participant stated:

‘I think in the hospital, even though they know how Jehovah’s Witnesses are, but because they don’t believe in that, sometimes you can see that they don’t take you seriously. Even though they’re not gonna do anything against your wishes, you can see they feel like you are risking.’ (MP 2, 43 year old, female)

The cultural misunderstandings were perceived to be judgemental. One participant shared:

‘I use herbs … But my problem is somebody whom is black, who knows what we as black people do but wants to come and discriminate or have a negative attitude towards that …’ (MP 1, 36 year old, female)

Understanding and respecting cultural beliefs play a major role in fostering trust and promoting the well-being of hospitalised. This underscores the need to institute measures to address the gaps in the provision of culturally sensitive nursing care. The findings from this conceptual phase were used to derive a central concept guided by Walker and Avant (2019). The findings from the first phase were used to develop the model.

#### Phase 2: Model development

The model was developed in four steps guided by Chinn et al. (2022): Step 1: Concept analysis, Step 2: Relationship statements, Step 3: Model development and Step 4: Model evaluation. The steps are described as follows:

**Step 1: Concept analysis:** Walker and Avant (2019) describe concept analysis as a rigorous process of examining the attributes and uses of concepts to enhance understanding and generate knowledge, resulting in precise definitions. The central concept has been derived from the findings of the first phase of the study. Walker and Avant’s concept analysis was used to systematically examine the phenomenon, identify its defining attributes, antecedents and consequences and thereby derive a clearly formulated central concept. The central concept derived was the facilitation of culturally sensitive practices in nursing care.

Facilitation is a dynamic process through which the nurse provides comprehensive support to transform clinical practice for better patient outcomes (Zhang et al. 2024). Facilitation, therefore, is a tool used by professional nurses to provide culturally sensitive care to patients. Through the facilitation of culturally sensitive practices in nursing care, the professional nurse actively, respectfully and compassionately engages with patients and considers their beliefs, traditions and cultural needs. The professional nurse engages in continuous learning about diverse cultural practices and interacts meaningfully with patients from diverse cultural backgrounds to facilitate the delivery of care. The dynamic interactive nature of the facilitation process implies that constant changes will occur, requiring the professional nurse to continuously adapt in response. Culturally sensitive practices in nursing care require meaningful interactions between the professional nurses and the patients during their shared moments of care.

The interrelatedness and interconnectedness between the central concept and its associated concepts were described to strengthen the model’s foundation.

**Step 2: Relationship statements:** Relationship statements are structural interconnections and interrelations articulated among the central concept and its associated concepts to provide insights into their complex dynamics (Chinn et al. 2022). Facilitating culturally sensitive nursing practices for children is a dynamic, interactive process that requires collaboration between the professional nurse and the patient to achieve the desired outcomes of cultural sensitivity. The assumptions of the model are grounded primarily on Madeleine Leininger’s Culture Care Theory, which emphasises that cultural diversity necessitates that professional nurses care for patients irrespective of their cultural backgrounds, beliefs and practices (Leininger 2008).

The relationship statements developed centre around four themes: challenges faced by the professional nurses, the mothers’ expectations, the need for collaboration and mobilisation of resources. Professional nurses face the challenge of being unable to facilitate culturally sensitive nursing care because of a lack of cultural competence skills. This causes frustration and distress, as it often leads to patients’ dissatisfaction. The mothers expect the professional nurses to know their children’s cultural beliefs and practices, as they believe this would improve care, increase satisfaction and possibly shorten hospital stays through collaboration in care. Effective facilitation of culturally sensitive nursing care requires collaboration between professional nurses and patients. The professional nurses need to mobilise and utilise resources and refer to institutional guidelines for effective facilitation of culturally contextual care for the children.

The relationship statements assisted in the development of the model.

**Step 3: Development and description of the model:** The Development and Description of the model were guided by the six elements of Dickoff et al. (1968) and six components of Chinn et al. (2022), ensuring its structural clarity and rigour. The six elements of Dickoff et al. (1968) not only provide the model with its structure but they also ensure that the model is operational for the daily facilitation of culturally sensitive nursing care. The components of Chinn et al. (2022) facilitated the description guidelines for operationalisation of the model. The model is distinct in that it places the paediatric patients at the centre of care, catering for their unique cultural and developmental needs.

The elements define who is involved in the model, the *agent* and the *recipient*; where the process occurs, *context*; what actions are needed for the process to occur, *procedure*; how the process is fuelled, *dynamics* and what the envisioned results are, *terminus* (Dickoff et al. 1968). The professional nurse is the primary agent in the model. The professional nurse has self-awareness and is committed to facilitating culturally sensitive nursing care. The primary recipient of care is the child with an illness. The child is in the legal care of the mother and the family. The mother and the family are therefore the secondary recipients of care. The context of the model is three layered. The outermost layer is the national healthcare framework, the middle layer is the children’s hospital and the innermost layer is the ward where the actual facilitation occurs. According to the South African Constitution, everyone, including the recipient, has the fundamental right to healthcare (Republic of South Africa 1996), which should be afforded at all costs. The context aims at protecting the rights of the child. The procedure is the facilitation of cultural knowledge. The professional nurse clarifies their own values and how they could impact healthcare. The driving power of the model – the dynamic – is the professional nurse’s awareness of their lack of cultural competence skills. The terminus is characterised by successful facilitation of culturally sensitive practices for nursing care. The professional nurse shows increased motivation and job satisfaction, as well as reduced frustration. Recipients of care have increased satisfaction and improved healthcare outcomes, as measured by discharge surveys.

### Assumptions of the model

The model is based on assumptions aligned with Madeleine Leininger’s Culture Care Theory, Campinha-Bacote’s Model of Cultural Competence and the Ubuntu philosophy. Leininger (2008) emphasises that caring is connected to culture because culture informs the individual’s healthcare expectations. Children’s perceptions and experiences of healthcare are shaped by their cultural beliefs and practices. Nurses are therefore expected to provide culturally appropriate and sensitive care for children from diverse cultural backgrounds (Tavallali 2025:75). The Culture Care Theory emphasises that, when healthcare is provided according to the cultural needs, the children develop lasting and trusting relations (Leininger 1988).

The model is based on seven assumptions summarised as follows:

Caring is culturally embedded, and this is based on Leininger’s view that care and culture are inseparable (Leininger 1997). Care, as conceptualised by Watson (2012), is defined as the foundation of nursing practice and is characterised by shared, meaningful moments between the professional nurses and the patients, including children. These shared moments are central to the facilitation of culturally sensitive practices for nursing care. It is during these moments that the professional nurse learns about the cultural beliefs and practices of the children, as well as their healthcare expectations. Respectful engagement enhances understanding, trust-building and mutual respect. Culture refers to the traditions and beliefs shared by individuals, families and communities.

Children’s healthcare experiences and cultural identities are shaped by their cultural backgrounds, and therefore, professional nurses must respond accordingly. The appropriate response requires professional nurses to be culturally competent. A culturally competent professional nurse has five skills of cultural awareness, cultural knowledge, cultural skill, cultural encounter and cultural desire (Campinha-Bacote 2002). The professional nurse clarifies their own values and becomes aware of various cultural beliefs and practices as they encounter children from diverse cultural backgrounds. Furthermore, the mothers mediate the children’s voices.

The professional nurse may initially lack cultural competence, which develops through reflection, education and encounters. This development is driven by the professional nurse’s awareness of skill gaps and commitment to learning. Ubuntu underpins the caring relationship and is the foundation of culturally sensitive nursing care. Ubuntu is seen through shared humanity, respect for human dignity and interconnectedness. Respectful engagement between the professional nurses and the mothers enhances mutual respect, understanding and the building of trust.

### The structure of the model

**FIGURE 1:**
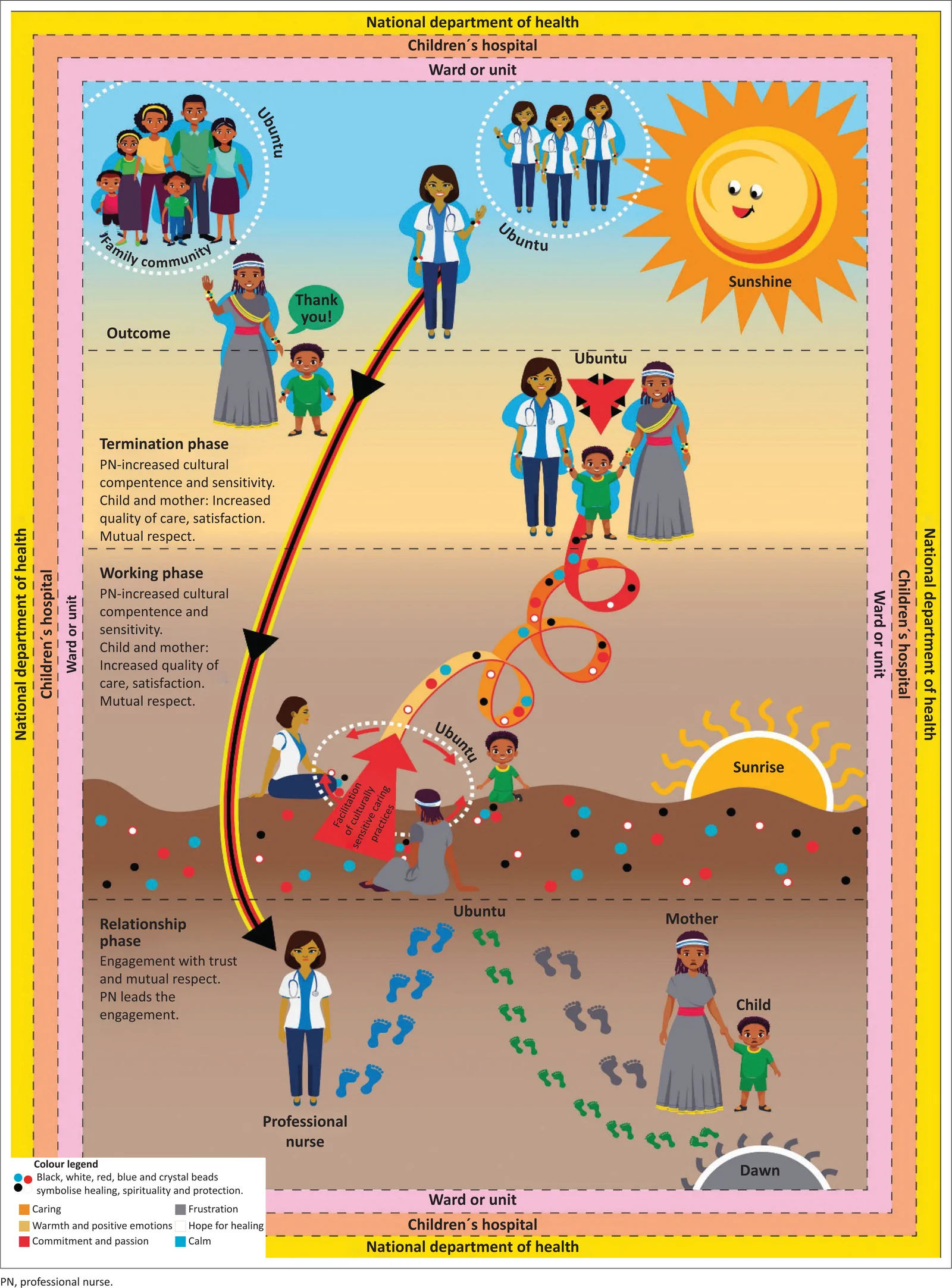
The structure of the model.

Structurally, the model is framed by the three-layer context that encloses the three phases of care and the outcome (Figure 1). The phases are inspired by Peplau’s Theory of Interpersonal Relationships: (1) the relationship phase, (2) the working phase and (3) the termination phase (Peplau 1997). These phases coincide with the patient’s hospitalisation journey. The relationship phase coincides with the admission process, the working phase with the hospitalisation stays and the termination phase with the patient’s discharge.

The model uses an African bead bracelet as a metaphor for the facilitation of culturally sensitive practices in nursing care. A metaphor is a figure of speech where a word is used to describe another unrelated word (Concise Oxford English Dictionary 2011) and to clarify processes (Steele, Baird & Davies 2022). Different colours of the African beads have been used to represent patients from diverse cultural backgrounds. The individual colours of the beads do not represent any specific cultural group; however, the differences among them serve to illustrate cultural diversity. The combination of the colours of the beads symbolises healing and protection (Jonauskaite & Mohr 2025). A combination of white, red, crystal and blue coloured beads is used.

The beads are assembled along a strong cord to create a bracelet, symbolising the facilitation process. The professional nurse selects the beads symbolising mobilisation and utilisation of resources for cultural care. The child and the mother join the professional nurse in selecting the beads symbolising collaboration, an essential aspect of culturally sensitive nursing care. The strong cord symbolises the difficulty of the facilitation process, necessitating trust, respect, understanding, compassion and care during the process. Assembling the beads requires careful selection of different beads. The facilitation process is driven by communication. The selection of the beads represents cultural assessment during the relationship phase; assembling the beads into a bracelet symbolises cultural care facilitation during the working phase; and the wearing of the beautifully made bracelet, the termination phase, requires effective communication and collaboration between the professional nurse, the child and the mother. This metaphor as an agent of change (Steele et al. 2022) symbolises the transformation of care to be more inclusive and sensitive to diverse cultural beliefs and practices.

Different colours have been used in the model’s structure to convey distinct cultural meanings (Jonauskaite & Mohr 2025). The context of the model is three layered, with the layers coloured orange, yellow and red, progressing from the outer to the inner layer. These colours represent the emotional tones of the hospital context. Orange represents the hospital as a healing environment (Jonauskaite & Mohr 2025). Yellow represents happiness, symbolising hope for successful facilitation of cultural care. Red symbolises the passion and commitment with which the professional nurse engages with the child and the mother in the ward. While the hospital is associated with negative emotions, the orange, yellow and red colours of the context show its positive side of hope for healing (Alzahrani 2021). The child and the mother join the family and the community represented by the white colour, embracing the outcome of healing (Ulusoy, Olguntürk & Aslanoğlu 2020). The facilitation process occurs in three phases, described next.

#### The relationship phase

The relationship phase lays the groundwork for trust and mutual respect. The professional nurse meets the child and the mother for the first time and uses communication and interpersonal skills to initiate cultural engagement (Abraham et al. 2024; Sherine et al. 2021). Peplau (1997) describes this as the orientation phase. The professional nurse lacks the skills needed for cultural competence, which drives the motivation and purpose behind the facilitation process. The professional nurse learns cultural competence skills and starts by clarifying their own values to prevent cultural misunderstandings and potential biases when providing cultural care. This occurs in the context of the children’s hospital. The professional nurse performs a cultural assessment. During the relationship phase, the sun appears to be gloomy at dawn because of the uncertainties. The mother has cultural care expectations for her child and is not sure whether they will be accommodated, and the professional nurse lacks cultural competence skills and is unsure as to how she will meet the cultural care needs. The dawn symbolises the beginning of the journey with the hope of sunrise and sunshine; hence, the professional nurse, the child and the mother are seen walking together into the working phase.

#### The working phase

The working phase is characterised by prolonged engagement and coincides with the hospitalisation stay. The soil symbolises life and provides a fertile ground for healing (McElwee 2021), epitomising the ground for the cultivation of knowledge. The professional nurse learns the skills needed to facilitate culturally sensitive nursing care. As the soil needs to be worked to yield a fruitful harvest, the professional nurse needs to actively learn and master the cultural competence skills of cultural awareness, cultural desire and cultural skill in preparation for cultural encounters.

The facilitation of culturally sensitive practices for nursing care is a cyclic process of applying the five cultural competence skills (Campinha-Bacote 2002). Cultural awareness enables the professional nurse to reflect on their own cultural beliefs and practices and how they could potentially create bias in care. The professional nurse assesses the cultural beliefs and practices of the child. They further assess the child’s healthcare expectations based on the cultural beliefs and practices to plan care. In planning this care, the professional nurse is mindful of their own cultural beliefs and constantly assesses if the care plan designed for the child is not compromised by their own cultural biases. Through cultural knowledge, the professional nurse can collect accurate and specific cultural data to ensure accommodation of the child’s cultural needs. The mastery of cultural skills prepares the professional nurse for meaningful future cultural encounters. This further motivates the professional nurse to develop cultural desire, a sense of readiness and the need to meet patients from diverse cultural backgrounds to facilitate culturally sensitive practices for nursing care.

The sunrise beams the rays of hope for this learning to be a success. The sun also provides the warmth needed for continued trust and understanding between the professional nurse, the child and the mother. This warmth also eases the tension and frustration from the relationship phase. Furthermore, the sun provides the energy (Yazev 2023) with which the professional nurse, the child and the mother interact. Facilitation requires collaboration and interconnectedness; hence, it is underpinned by Ubuntu.

The professional nurse mobilises and utilises resources to facilitate cultural care. These include enrolling for an upskilling training programme on culturally sensitive nursing care, referring to institutional policies and guidelines on cultural sensitivity when providing care to the children and involving other members of the multidisciplinary team. The professional engages in continuous reflection of the cultural care they are providing, constant evaluations by the recipients and immediate improvements.

### Ethical considerations

Ethical clearance was obtained from the University of Johannesburg’s Research Ethics Committee (REC-821-2020), and the healthcare facility provided written permission for the study to be conducted. The fundamental ethical principles of autonomy, non-maleficence, beneficence and justice were adhered to throughout the study (Dhai & McQuoid-Mason 2020). Informed consent was obtained from all the participants prior to participating in the study, with the understanding that they could withdraw anytime without penalty.

## Results

The results of the facilitation process are seen in the termination phase.

### The termination phase

The termination phase is the last phase of the patient care journey and is realised when the goals have been achieved at the point of discharge. Effective facilitation of culturally sensitive practices for nursing care results in positive professional outcomes, including enhanced job satisfaction, strengthened cultural competence, reduced frustration and increased cultural desire for the professional nurse. For the child, the termination phase is associated with improved clinical outcomes and greater levels of satisfaction, while the mother experiences a sense of relief, reassurance and overall satisfaction with the cultural care provided to the child.

### The outcome

The professional nurse becomes confident in cultural competence skills and desires subsequent encounters to further engage in and apply these skills. There is increased job satisfaction and the desire to contribute to the development of others. Facilitation occurs through a feedback loop across the model; therefore, should there be a relapse, the professional nurse is able to revisit the process at the appropriate phase. There is an improved quality of care and greater satisfaction for the child, the family and the larger community. They feel respected and understood. The hospital stay potentially becomes shorter because of improved cooperation and trust. Through gaining trust in the healthcare system, the community utilises the healthcare services with trust.

### Step 4: Evaluation of the model

The model was evaluated by nursing theory experts in qualitative research and theory generation. The demographics of the experts are provided in Table 4. The model evaluation sought the experts’ critical reflections on five aspects: (1) clarity, (2) simplicity, (3) generality, (4) accessibility and (5) importance of the model (Chinn et al. 2022).

**TABLE 4:** Demographics of the experts who evaluated the model.

Expert panellists (EP)	Highest qualification	Current job title	Experience in qualitative research (years)	Experience in knowledge generation (years)
1	PhD in Nursing	Professor	18	Not own specialty; however, well-read.
2	DCur	Associate Professor	11	11
3	PhD	Associate Professor	10+	10+
4	PhD	General Manager and Midwife Specialist	10	4
5	PhD in Nursing	Midwifery Lecturer	8	1
6	PhD in Nursing Sciences	Senior Lecturer and Advanced Midwife specialist	8	5
7	PhD	Senior Lecturer	6	4

PhD, doctor of philosophy; DCur, doctor of nursing science.

The feedback reflected high levels of clarity and perceived importance, with constructive suggestions to improve accessibility and universality. The model is believed to be clear:

‘Very clear. The colours and story are clear.’ (EP3, PhD, Associate Professor)

The feedback also highlighted the simplicity of the model as expressed by one expert:

‘The model is appropriately simple, providing a structured framework that captures essential elements of culturally sensitive nursing without being overwhelming.’(EP7, PhD, Senior Lecturer)

With respect to the applicability of the model, one expert commented as follows:

‘The model is applicable to be used within a Pan-African context. Beads are worn and carry cultural significance throughout countries where Black Africans make up the indigenous population. Furthermore, the history of colonialism in Africa has led to the dominance of Western medicine and a Western way of knowing which has displaced Indigenous Knowledge Systems within the health care system. Thus, the model can be used in different settings in Africa to inform cultural sensitivity and foster a bridge between the health care workers and the families they care for.’ (EP5, PhD in Nursing, Midwifery Lecturer)

One expert highlighted the model’s importance by saying:

‘This is an important model as it addresses a critical aspect of nursing. SA is known for its diversity and the nursing profession in South Africa is the most relevant space to develop such a model to model for the rest of the world.’ (EP2, DCur, Associate Professor)

The model is said to be accessible within the healthcare and cultural context:

‘The model is highly accessible to professional nurses, as it uses clear, practical concepts and guidance that do not require advanced theoretical expertise. Its structure and tools are designed to be easily implemented in daily practice, facilitating widespread adoption and consistent application of culturally sensitive care.’ (EP7, PhD, Senior Lecturer)

Overall, the experts agreed that the model demonstrated conceptual clarity, practical simplicity, theoretical generality and contextual accessibility. The model integrated theoretical and regulatory representation, colour coding and the strong underpinning by Ubuntu philosophy.

The model needs to be used effectively to realise the desired outcomes. Evaluation of the model led to the development of the following guidelines for its operationalisation.

#### Guideline 1: Relationship phase

The relationship phase coincides with the admission of the patient, and during this phase, the professional nurse needs to lay the groundwork for trust, understanding and respect for meaningful cooperation and collaboration. As it is a period of brief engagement, the professional nurse needs to engage in honest reflection on their own cultural values, beliefs and practices to clarify their own values. Value clarification fosters self-awareness, an essential trait to prevent cultural misunderstandings. It is essentially during this phase that the professional nurse creates a positive and conducive environment for cultural care. Communication facilitates the creation of this positive environment.

#### Guideline 2: Working phase

The working phase coincides with the hospitalisation of the child. This is often lengthy, as the child could be admitted for a few days or longer. The working phase, therefore, is a period of prolonged engagement. The working phase is characterised by the actual facilitation process and mobilisation of resources. The professional nurse learns and applies the cultural competence skills of cultural awareness, cultural knowledge, cultural skills, cultural encounters and cultural desires to provide culturally sensitive nursing care. The mother shares cultural beliefs, cultural practices and cultural care expectations, which the professional nurse then integrates into the provision of care. The professional nurse, the child and the mother engage in collaboration. The professional nurse mobilises resources by leading cultural care discussions. There is a need for healthcare institutions to develop policies to guide professional nurses to facilitate culturally sensitive nursing care.

#### Guideline 3: Termination phase

During the termination phase, the attainment of the objectives of the relationship and the working phases are evaluated. The child and the mother reflect on the care they received and provide feedback on their experience. The professional nurse reflects on their own confidence in facilitating culturally sensitive practices for nursing care.

## Discussion

### Limitations

The study had three major limitations, namely sample limitation, researcher bias and implementation of the model. Professional nurses were sampled in the study, thereby excluding the enrolled nurses and enrolled nursing assistants. The experiences of these nursing categories have not been explored. The study was conducted at one children’s hospital; therefore, the experiences of the professional nurses and the mothers from the other children’s hospital and other hospitals with paediatric units have not been explored. In addition, even though the guidelines for the implementation of the model have been developed, the model has not been implemented. It is therefore recommended that future research should focus on including other categories of nurses, using stratified sampling strategies to include other hospitals providing paediatric care and the implementation of the model.

## Conclusion

Facilitation of culturally contextual nursing care remains a challenge, especially for children whose healthcare expectations are shaped by their cultural beliefs and practices. This challenge necessitated the development of a model to provide a practical guide for professional nurses to facilitate culturally sensitive nursing care practices for children. The professional nurses and the mothers emphasised the cultural care challenges of the dilemmas of misunderstood and unmet cultural expectations. A model was subsequently developed, described and evaluated. Accordingly, the model offers a practical solution to accommodating cultural beliefs and practices in healthcare.
